# Effects of warm ischemia and reperfusion on the liver microcirculatory phenotype of rats: underlying mechanisms and pharmacological therapy

**DOI:** 10.1038/srep22107

**Published:** 2016-02-24

**Authors:** Diana Hide, Martí Ortega-Ribera, Juan-Carlos Garcia-Pagan, Carmen Peralta, Jaime Bosch, Jordi Gracia-Sancho

**Affiliations:** 1 Barcelona Hepatic Hemodynamic Lab. IDIBAPS Biomedical Research Institute – Hospital Clinic de Barcelona – CIBEREHD. Barcelona, Spain; 2 IDIBAPS & CIBEREHD.

## Abstract

Warm ischemia and reperfusion (WIR) causes hepatic damage and may lead to liver failure, however the mechanisms involved are largely unknown. Here we have characterized the microcirculatory status and endothelial phenotype of livers undergoing WIR, and evaluated the use of simvastatin in WIR injury prevention. Male Wistar rats received simvastatin, or vehicle, 30 min before undergoing 60 min of partial warm ischemia (70%) followed by 2 h or 24 h of reperfusion. Hepatic and systemic hemodynamics, liver injury (AST, ALT, LDH), endothelial function (vasodilatation in response to acetylcholine), KLF2 and nitric oxide pathways, oxidative stress, inflammation (neutrophil and macrophage infiltration) and cell death were evaluated. Profound microcirculatory dysfunction occurred rapidly following WIR. This was evidenced by down-regulation of the KLF2 vasoprotective pathway, impaired vasodilatory capability and endothelial activation, altogether leading to increased hepatic vascular resistance and liver inflammation, with significant leukocyte infiltration, oxidative stress and cell death. Simvastatin preserved the hepatic endothelial phenotype, and blunted the detrimental effects of WIR on liver hemodynamics and organ integrity. In conclusion, WIR-induced injury to liver sinusoidal endothelial cells is mitigated by pre-treatment with Simvastatin probably through a KLF2-dependent mechanism.

Ischemia/reperfusion injury is the phenomenon of interruption of blood supply followed by restoration of blood flow and the accompanying oxygen, nutrient supply and shear stress. Clinically, warm ischemia/reperfusion (WIR) injury is almost unavoidable in liver resection surgery, liver transplantation, and in blood transfusion for hemorrhagic shock, and may lead to delayed graft function and liver failure. Two different phases can be distinguished during reperfusion: an early phase (within 2 h after restoring reperfusion), which is characterized by the release of reactive oxygen species (ROS) and production of inflammatory mediators (TNFα, chemokines)^1^,^2^, and a late phase (6–48 h after reperfusion), in which inflammatory responses caused by neutrophil and macrophage infiltration exacerbate the liver damage^3^. As demonstrated recently by our group, the effects of ischemia reperfusion are already significant under cold ischemia when further deterioration of hepatic microcirculation and endothelial dysfunction is seen^4^.

Kruppel-like factor 2 (KLF2) is a transcription factor predominantly expressed by the endothelial cell^5^ that induces expression of vasodilator, anti-thrombotic and anti-inflammatory genes (e.g. endothelial nitric oxide synthase (eNOS) and thrombomodulin)^6^,^7^ and inhibits the expression of adhesion molecules (vascular cell adhesion molecule 1 (VCAM-1) and E-selectin)^6^,^8^ maintaining a vasoprotective endothelial phenotype. Experimental studies using endothelial cells have demonstrated that KLF2 expression is induced by physiological blood flow-derived shear stress^9^,^10^.

Statins are HMG-CoA inhibitors originally designed to lower cholesterol levels, however they have shown other therapeutic effects independent on lipid lowering^11^. Administration of simvastatin in experimental models of chronic and acute liver injury has demonstrated its effectiveness in protecting the liver sinusoidal endothelium^12^,^13^,^4^, nevertheless its potential applicability in situations of hepatic WIR is unknown. These beneficial effects of statins are due in part to the activation of KLF2 pathway that contributes in maintaining/restoring a healthy endothelial phenotype^14^,^15^.

In the present study we have characterized for the first time the hepatic microcirculatory status and sinusoidal endothelial phenotype of livers undergoing warm ischemia and reperfusion injuries, and evaluated the applicability of simvastatin to improve or prevent warm ischemia/reperfusion injury.

## Results

### WIR causes microcirculatory derangements and endothelial dysfunction

**Figure 1:**
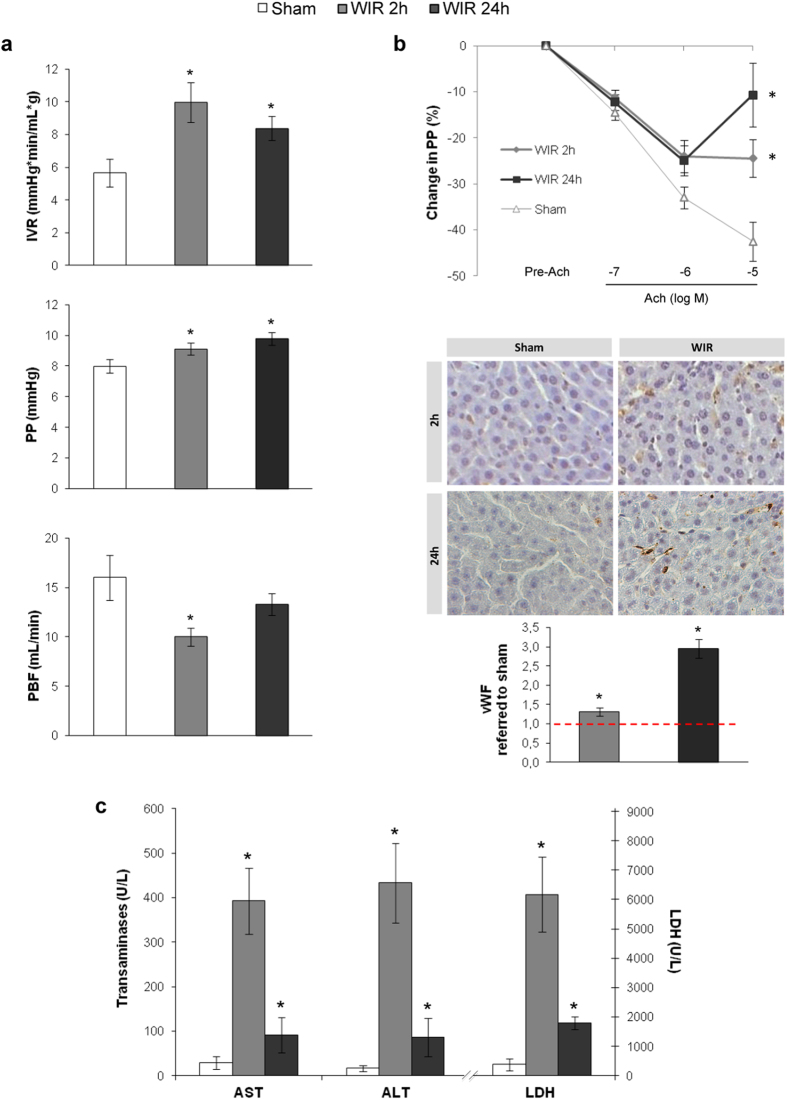
Liver warm ischemia and reperfusion leads to acute hepatic microvascular dysfunction. (**a)** Intrahepatic vascular resistance (IVR), portal pressure (PP) and portal blood flow (PBF) determined in rats that underwent 70% hepatic warm ischemia followed by 2 h or 24 h of reperfusion (WIR). **(b)** Hepatic endothelial function evaluation analyzing relaxation response to incremental doses of Ach (top) and vWF protein expression (down) in rats described in A. Representative images (40 × magnification). **(c)** Hepatic injury evaluated as release of transaminases (ALT, AST) and LDH in liver perfusate from rats described in A. (n = 8 per group; *p < 0.05 vs. sham).

Livers undergoing warm ischemia and reperfusion (WIR) exhibited significantly impaired microcirculation evidenced by a marked increase in intrahepatic vascular resistance at 2 h and 24 h of reperfusion, associated with increased portal pressure and a reduction of portal blood inflow (Fig. 1a). Furthermore, WIR promoted the development of acute endothelial dysfunction defined as a reduced response to the endothelial-dependent vasodilator acetylcholine (Fig. 1b) and an increase in the sinusoidal expression of the liver sinusoidal endothelial cells (LSEC) capillarization marker von Willebrand Factor (vWF, Fig. 1b). The detrimental effects of WIR on liver endothelial function observed at 2 h of reperfusion were exacerbated after 24 h (Fig. 1b).

In addition, WIR caused a significant increase in the release of transaminases and LDH compared to sham operated animals especially at the early phase of reperfusion (Fig. 1c). At 24 h of reperfusion these parameters were decreased but still were significantly higher than in sham animals.

### WIR reduces KLF2 expression and NO bioavailability

**Figure 2:**
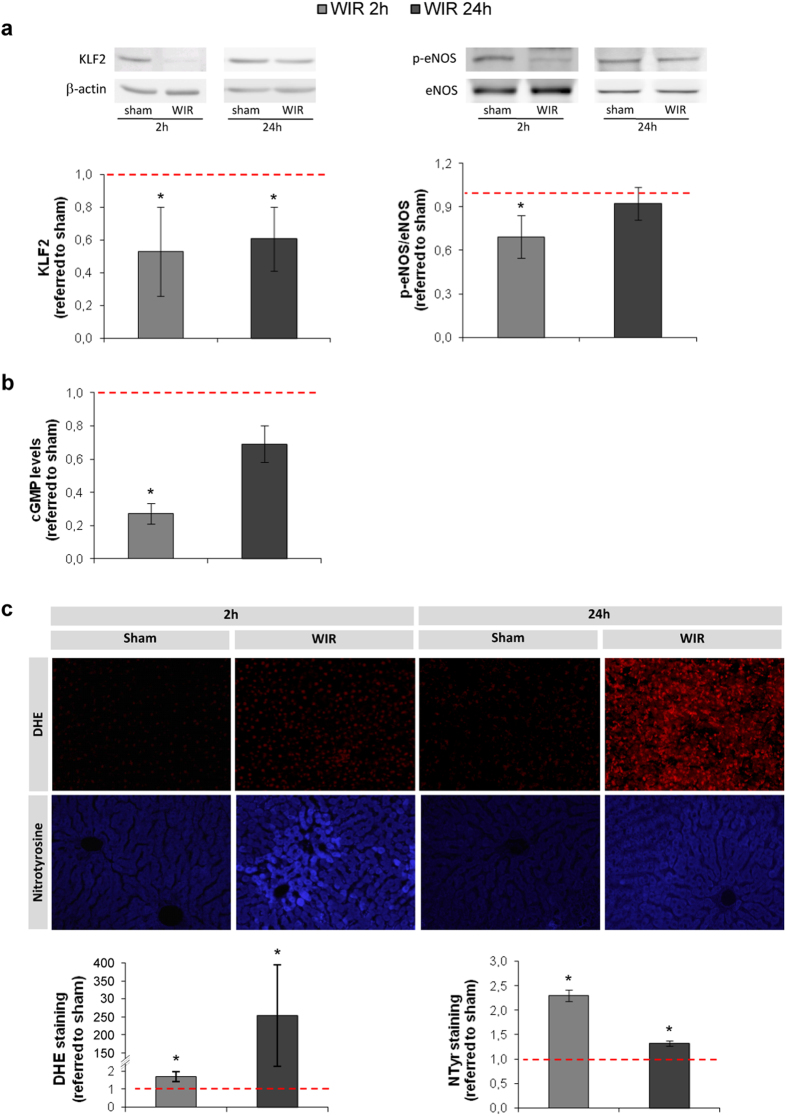
Hepatic warm ischemia and reperfusion induces a rapid down-regulation in KLF2 and its derived vasoprotective programs. (**a)** Representative images of hepatic KLF2, phosphorylated eNOS (p-eNOS) and total eNOS immunoblots and densitometric analysis normalized to β-actin from rats undergoing 1 h of partial ischemia followed by 2 h or 24 h of reperfusion (WIR), compared to the sham group. **(b)** cGMP levels in livers described in A. **(c)** Representative images and quantitative analysis of DHE staining and nitrotyrosinated proteins fluorohistochemistry (20 × magnification) (n = 8 per group; *p < 0.05 vs. sham). The red dotted line represents the sham group.

WIR caused a reduction in KLF2 liver protein expression at the early and late phases of reperfusion (Fig. 2a left), which was accompanied by a decrease in phosphorylated eNOS (Fig. 2a right) and a diminution in NO bioavailability (Fig. 2b). O_2_^−^ production and nitrotyrosine formation (caused by the scavenging of NO by O_2_^−^) were significantly increased at both time points of reperfusion (Fig. 2c).

### WIR promotes hepatic inflammation and cell death

**Figure 3:**
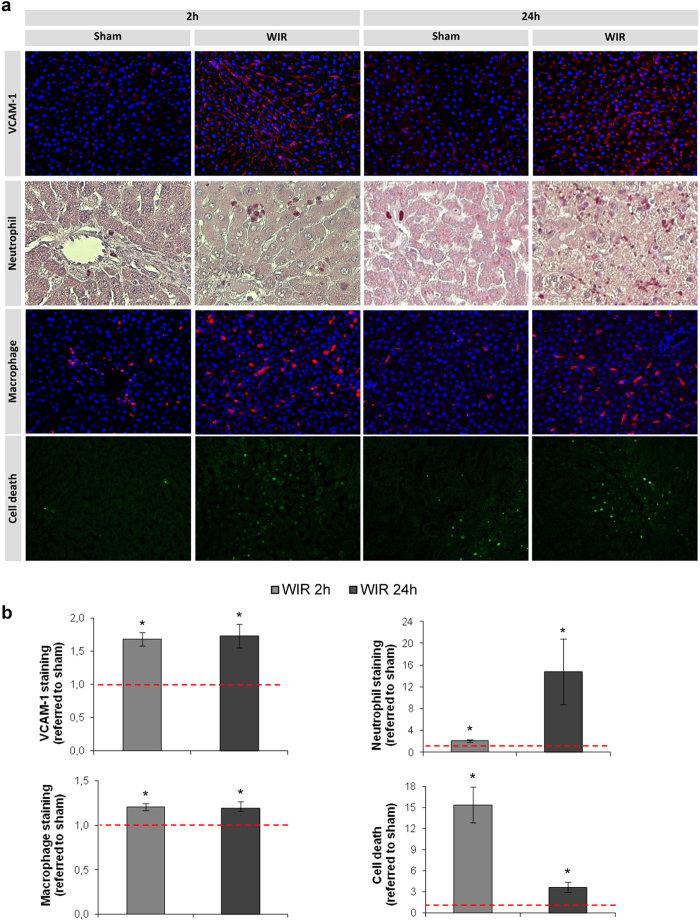
Liver warm ischemia and reperfusion promotes endothelial cell activation, leukocyte infiltration and cell death. **(a)** Representative images of VCAM-1 and CD68 immunohistochemistry, neutrophil infiltration and TUNEL staining determined in livers from rats that underwent 1 h of partial hepatic ischemia followed by 2 h or 24 h of reperfusion (WIR) (neutrophil magnification 40×, other 20×); **(b)** quantitative analysis of at least 8 fields/sample referred to its sham group (red dotted line). (n = 8 per group, *p < 0.05 vs. sham).

WIR injury induced a rapid activation of the hepatic endothelium, as demonstrated by the increases in P-Selectin (5.8 fold and 2.3 fold increments at 2 h and 24 h, respectively, p < 0.05) and VCAM-1 (Fig. 3), which was associated with subsequent infiltration and accumulation of macrophages and, especially, neutrophils within the liver parenchyma (Fig. 3). In addition, terminal deoxynucleotidyl transferase dUTP nick end labeling (TUNEL) staining revealed that there was a significant 15-fold increase in cell death after 2 h of reperfusion, which was partly resolved at 24 h but remaining significantly higher than in sham operated animals (Fig. 3).

### Simvastatin improves WIR-mediated liver microcirculatory dysfunction

**Figure 4:**
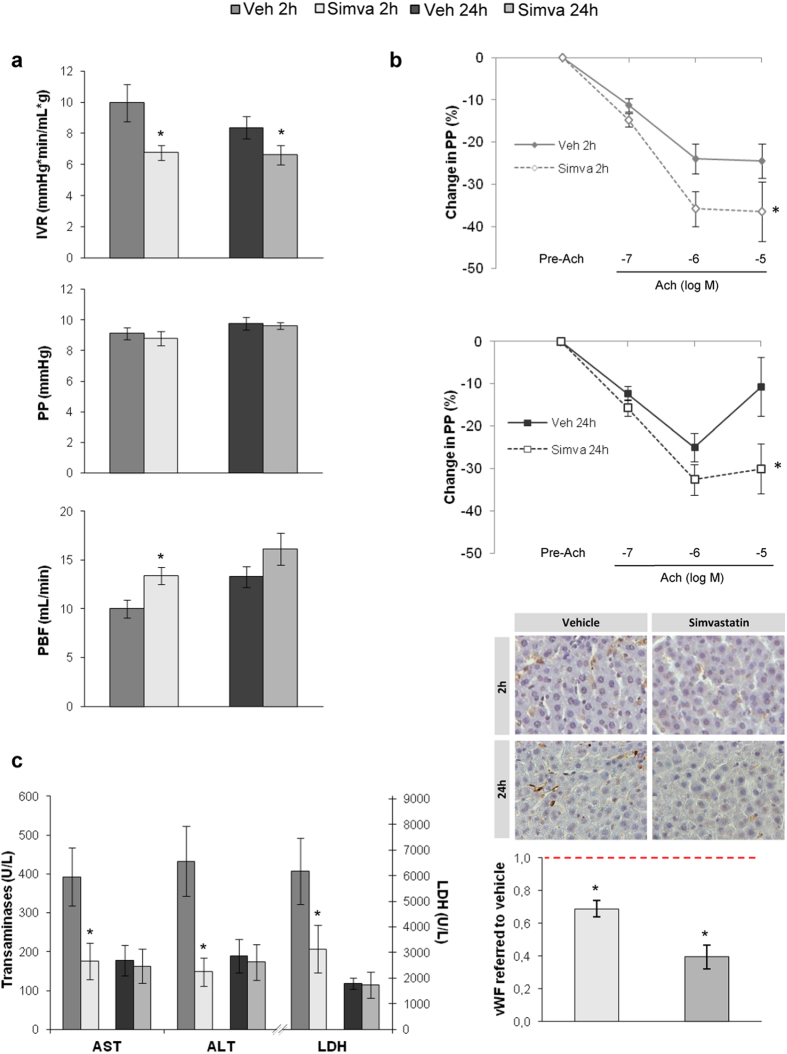
Simvastatin prevents liver vascular dysfunction produced by warm ischemia and reperfusion injury. (**a)** Intrahepatic vascular resistance (IVR), portal pressure (PP) and portal blood flow (PBF) in rats pre-treated with vehicle or simvastatin and afterwards undergoing liver warm ischemia and reperfusion. **(b)** Endothelial function evaluation (top) and vWF immunohistochemistry (down) in animals described in A (40 × magnification), normalized to its corresponding vehicle-treated group (red dotted line). **(c)** Hepatic injury evaluation. (n = 8 per group; *p < 0.05 vs. vehicle).

Simvastatin administration 30 minutes before ischemia prevented the WIR-derived increment in intrahepatic vascular resistance, thus improving liver perfusion without changes in portal pressure (Fig. 4a). In addition, simvastatin significantly attenuated the development of acute endothelial dysfunction both at 2 and 24 h of reperfusion as evidenced by improved vasodilation in response to Ach (Fig. 4b top) and reduced sinusoidal vWF expression (Fig. 4b bottom). The improvement in hepatic hemodynamics was associated with a much less marked increase in transaminases and lactate dehidrogenase (LDH) release (Fig. 4c).

### Simvastatin prevents KLF2 down-regulation and maintains NO bioavailability

**Figure 5:**
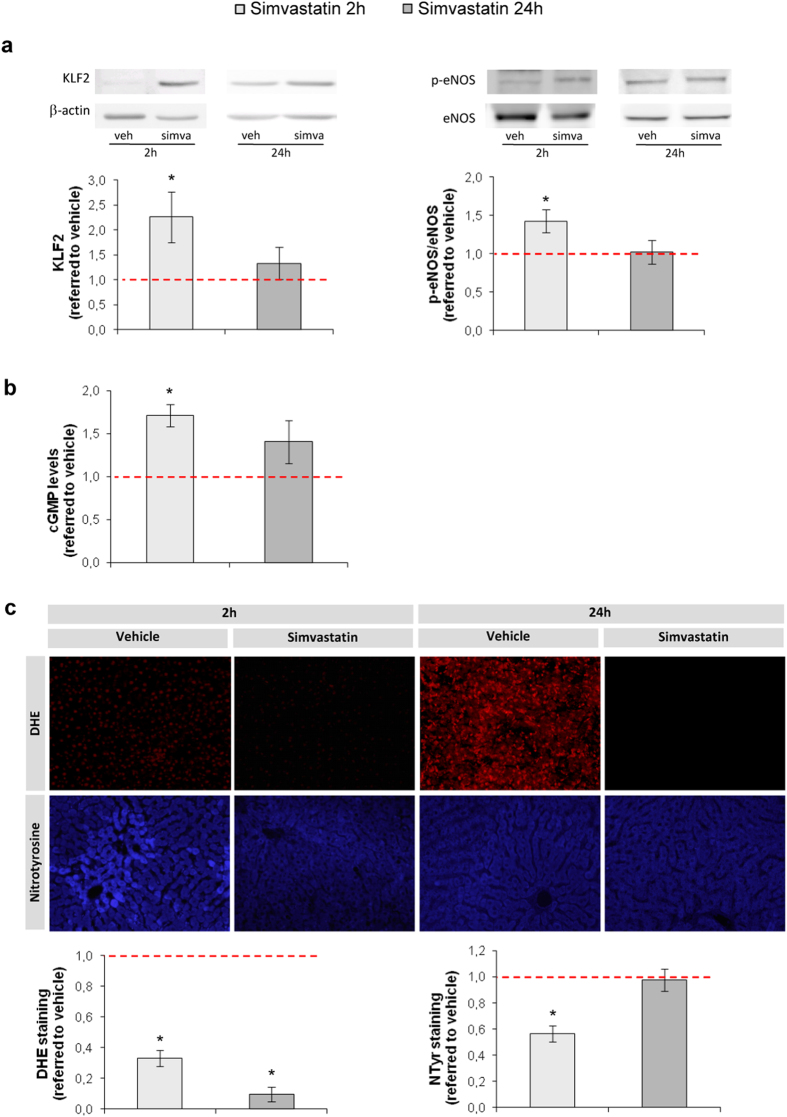
Simvastatin pre-treatment maintains the KLF2-derived vasoprotection during warm ischemia and reperfusion injury. (**a)** Representative images of hepatic KLF2, p-eNOS and eNOS immunoblots and densitometric analysis normalized to β-actin. **(b)** Hepatic cGMP levels. **(c)** Representative images and quantitative analysis of DHE staining and nitrotyrosinated proteins fluorohistochemistry (20×). All quantifications normalized to its corresponding vehicle (red dotted line). (n = 8 per group; *p < 0.05 vs. vehicle).

Administration of simvastatin prior to ischemia maintained liver KLF2 protein expression and eNOS phosphorylation levels, which was especially evident at 2 h of reperfusion (Fig. 5a). This was accompanied by higher NO bioavailability (Fig. 5b), and by decreased O_2_^−^ production and O_2_^−^-mediated NO scavenging (Fig. 5c).

### Simvastatin administration inhibits endothelial activation, inflammation and cell death

**Figure 6:**
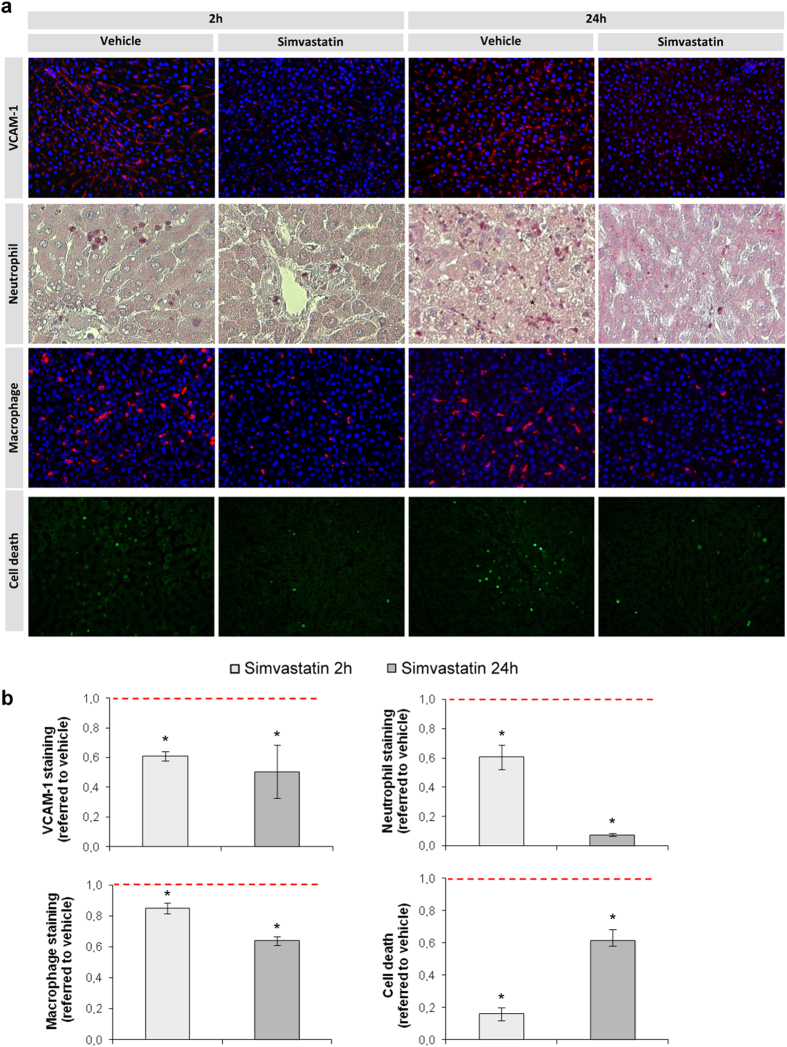
Simvastatin reduces hepatic endothelial activation, leukocyte infiltration and cell death derived from warm ischemia and reperfusion injury. Representative images of VCAM-1, neutrophil and macrophage infiltration, and cell death detection in livers from rats pre-treated with simvastatin, or its vehicle, before undergoing 1 h of warm ischemia followed by 2 h or 24 h of reperfusion (**a**) and its corresponding quantifications (**b**). Neutrophil 40×, other 20×, values normalized to its vehicle group (red dotted line). (n = 8 per group, *p < 0.05 vs. vehicle).

Liver endothelial phenotype maintenance due to simvastatin administration prevented an increased expression in adhesion molecules (P-Selectin 55% lower at 2 h of reperfusion, and 98% at 24 h vs. vehicle-treated rats, p < 0.05; VCAM-1 Fig. 6), and neutrophil and macrophage infiltration (Fig. 6), both at the early and late phases of reperfusion. Moreover, simvastatin-treated animals showed a significant reduction in hepatic cells death (Fig. 6).

### Simvastatin increases KLF2 and improves the phenotype of hepatic cells *in vitro*

Simvastatin increased KLF2 mRNA expression not only in LSEC but also in primary hepatocytes and Kupffer cells (Supplementary Fig. S1a). This correlated with a slight improvement in the hepatocyte phenotype observed by a trend to an increase in the transcription factor HNF4α and a reduction in the Mrp3 transporter expression (Supplementary Fig. S1b). Regarding Kupffer cells, simvastatin treatment induced a polarization towards an anti-inflammatory M2 phenotype, evidenced by significant increases in the expression of Arg-1 and IL-10, without significant changes in the markers of M1 phenotype TNF-α and iNOS (Supplementary Fig. S1c).

LSEC treated with simvastatin showed a statistically significant 8-fold and 2.6-fold increase in KLF2 and eNOS mRNA expression respectively after reperfusion. This was associated with a decrease in the mRNA expression of the pro-inflammatory & capillarization markers iNOS (1.00 ± 0.16 vs. 0.76 ± 0.19) and VCAM-1 1.00 ± 0.06 vs. 0.85 ± 0.14). Furthermore, LSEC pre-treated with simvastatin exhibited lower levels of oxidative stress, measured as O_2_^−^, compared with vehicle-treated cells (1.00 ± 0.18 vs. 0.72 ± 0.18; p = 0.1).

## Discussion

Liver sinusoidal endothelial cells (LSEC) damage following ischemia reperfusion (IR) injury is the first event in the development of graft failure. Although this was already suggested in the late 1980 s^16^, most research to date has focused on other liver cell types. It is accepted that after IR there is an increase in LSEC ROS, together with an imbalance between decreased NO bioavailability (due to reduced production by eNOS and increased scavenging by ROS) and increased endothelin and thromboxane A2 levels, altogether promoting the expression of adhesion molecules, neutrophil adhesion and platelet aggregation^17^,^18^,^19^. However elucidation of the underlying mechanisms of LSEC damage during hepatic IR injury, and its impact on the global reduction in organ viability and function after liver resection or transplantation is scarce. In this work we have characterized the hepatic microcirculation in a well-established model of warm ischemia and reperfusion of the liver, focusing predominantly on changes in LSEC phenotype, its mechanisms, consequences and prevention.

In the present study, we demonstrate for the first time that a short period of warm ischemia is enough to cause striking deleterious effects on the hepatic microcirculation markedly increasing the intrahepatic vascular resistance (IVR), both at the early and late phases of reperfusion, leading to increased portal pressure and to a significant reduction in hepatic perfusion. Furthermore, animals suffering WIR develop acute endothelial dysfunction evidenced by a decreased response to the endothelial dependent vasodilator acetylcholine^20^ and by increased expression of vWF^21^,^22^. In addition WIR caused liver damage, measured as increments in AST, ALT and LDH, as described previously^23^,^24^, especially at 2 h of reperfusion. At the late phase of reperfusion, levels of transaminases and LDH seem to improve most likely due to an initial resolution of liver injury; nevertheless they remain significantly higher than in sham-operated animals.

It has been described that blood flow-derived shear stress maintains a vasoprotective endothelial phenotype owing to the activation of the transcription factor KLF2, which mediates the transcription of several protective genes^8^,^9^,^25^. Indeed, recent studies have demonstrated that lack of the biomechanical stimulus determined by shear stress deteriorates the endothelial phenotype by down-regulating the expression of KLF2^4^,^10^. Thus, we hypothesized that during WIR blood inflow blockade may also decrease liver endothelial KLF2 expression, and this reduction will cause a loss of liver vasoprotection during reperfusion.

To evaluate our hypothesis we measured KLF2 protein expression in liver tissue observing a significant reduction after WIR, both at the early and the late phases of reperfusion. Interestingly, this decrease was accompanied by a reduction in eNOS activation, one of KLF2 target genes. We further evaluated the bioavailability of the vasodilator NO by measuring the levels of cyclic guanosine monophosphate (cGMP) as a second messenger of NO and found a significant reduction in cGMP levels at the early phase of reperfusion. The reduction in NO bioavailability is explained by a combination of two factors: less synthesis due to reduced eNOS activity and; increased NO-scavenging by the high amount of O_2_^−^ produced during reperfusion. This scavenging causes the formation of the free radical peroxynitrite that may rapidly react with cell components such as proteins, lipids, and DNA further damaging the cell^26^.

We further characterized the consequences of liver endothelial de-regulation during WIR by analyzing liver inflammation at two reperfusion time-points: early (2 h) and late (24 h). We evaluated hepatic VCAM-1 and P-selectin protein expression due to their implication in neutrophil adhesion and extravasation^27^,^28^,^29^ and found an up-regulation at early and late reperfusion periods. Indeed, neutrophil infiltration was slightly increased after 2 h of reperfusion and greatly increased at 24 h. Furthermore macrophage activation and infiltration was also significantly increased at both reperfusion times. As it has been described, leukocytes produce pro-inflammatory cytokines and ROS further contributing to tissue damage^30^,^31^, this can also be observed in our results, with a vast O_2_^−^ production at the late reperfusion phase. Microvascular dysfunction and parenchymal damage associated with WIR induced hepatic cell death.

Statins or HMG-CoA inhibitors were primarily designed to decrease cholesterol levels but they have also shown to have beneficial cholesterol-independent effects such as up-regulating KLF2-derived transcriptional programs that improve endothelial function^14^,^32^. Considering the detrimental effects of WIR on liver microcirculation, KLF2 vasoprotective cascades, and recent data from our group demonstrating the beneficial effects of simvastatin in the cirrhotic liver^33^, we have applied an acute pharmacological pre-treatment with simvastatin to preserve liver endothelial function during WIR. Some previous experimental studies have applied statins to WIR and have shown benefits in terms of organ injury and inflammation^34^,^35^,^36^,^37^,^38^, however they have not evaluated the effects of statins on liver microvascular function and phenotype, especially when administered shortly before ischemia, thus discarding possible concomitant effects of lipid-lowering.

Our results demonstrate that simvastatin administration 30 min before ischemia prevents the increase in hepatic vascular resistance during reperfusion, and that is associated with better liver perfusion at early and late reperfusion periods. Simvastatin pre-treatment also improved liver endothelial function and prevented the peak in transaminases and LDH release observed at 2 h of reperfusion.

Evaluation of the KLF2 pathway revealed that simvastatin is able to prevent liver KLF2 protein expression decay observed at the early reperfusion period, thus maintaining the activation of its target gene eNOS, enhancing NO bioavailability. Moreover, simvastatin-treated animals showed reduced levels of ROS. This might be explained by the antioxidant properties of KLF2 through activation of nuclear factor (erythroid-derived 2)-like 2 (Nrf2) and up-regulation of heme oxygenase 1 (HO-1)^39^,^33^. Finally, we found that simvastatin prevented the expression of adhesion molecules such as VCAM-1 and P-selectin on the endothelial surface reducing leukocyte infiltration, which may also be attributed to the anti-inflammatory properties of KLF2^6^,^40^.

The results obtained *in vivo* were validated *in vitro*. LSEC cultured in an anoxia/reoxygenation system showed that simvastatin was able to partly abrogate the burst in oxidative stress and inflammation due to I/R, most probably due to the induction of the KLF2 protective pathway^10^. Furthermore, we herein demonstrate for the first time that simvastatin also activates the KLF2 pathway in other hepatic cells (ie. Hepatocytes and Kupffer cells), in which it also confers a protective phenotype. These observations complement previous data demonstrating the beneficial effects of statins in another non-parenchymal cell type, the hepatic stellate cells^33^, altogether reinforcing the concept of global hepatic protection in response to statins.

Despite the positive results achieved in this study using simvastatin in the setting of WIR injury, we have to acknowledge some limitations. First, the model of partial ischemia without hepatic resection, although representative of the WIR that occurs *in vivo* after episodes of shock and/or hypovolemia, does not allow extrapolating what happens in liver resection surgery. On the other hand, the role of KLF2 in protection against liver WIR could be further delineated using genetic models where KLF2 expression would be knocked-down specifically.

In summary, the results herein reported shed light on the pathophysiology of liver ischemia reperfusion injury demonstrating that a warm ischemia period, even of short duration, profoundly affects the liver endothelial phenotype, which becomes dysfunctional, leading to an immediate increase in the hepatic vascular tone, inflammation, polymorphonuclear cells infiltration, and hepatic cell death. WIR-derived damage to the hepatic endothelium may be, at least in part, due to KLF2 down-regulation. Indeed, simvastatin pre-treatment maintains KLF2 vasoprotective pathways and efficiently protects the hepatic microcirculation from WIR and prevents subsequent liver injury. Our data strongly suggest that simvastatin administration may represent an effective and simple approach to prevent or reduce liver damage in clinical situations associated with warm ischemia-reperfusion liver injury.

## Methods

### Animals and treatment

Male Wistar rats from Charles River Laboratories (Barcelona, Spain) weighting 300–350 g were used. Rats were treated with simvastatin (1 mg/kg i.v., Calbiochem, San Diego, CA) or its vehicle (DMSO 0.1%) 30 minutes before ischemia. Animals were kept in environmentally controlled animal facilities at the Institut d’Investigacions Biomediques August Pi i Sunyer (IDIBAPS). All experiments were approved by the Laboratory Animal Care and Use Committee of the University of Barcelona and were conducted in accordance with European Community guidelines for the protection of animals used for experimental or other scientific purposes (EEC Directive 86/609).

### Liver Vascular Studies

Under anesthesia with intraperitoneal ketamine (100 mg/Kg, Merial Laboratories, Barcelona, Spain) and midazolam (5 mg/Kg, Normon, Tres Cantos, Madrid, Spain) partial warm ischemia affecting 70% of liver volume was induced by clamping the portal triad irrigating the medial and left lateral lobes with an atraumatic clamp for 1 h^41^, which was followed by 2 h (short-term) or 24 h (long-term) of reperfusion. Sham operated animals were included. After the WIR period, hemodynamic studies were performed as previously described^42^. Briefly, a tracheotomy was performed and a polyethylene PE-240 tubing was inserted into the trachea to ensure a patent airway. PE-50 catheters were introduced into the femoral artery, for arterial pressure recording (mm Hg), and into the portal vein through the ileocolic vein, to measure portal pressure (mmHg). Then, the portal vein was carefully dissected free from connective tissue, and a non-constrictive perivascular transit-time ultrasonic flow probe (Transonic Systems, Ithaca, New York, USA) was placed around the vessel. The flow probe was connected to a flow-meter, to measure the portal vein blood flow (mL/min). Intrahepatic vascular resistance (IVR; mmHg/mL·g·min^−1^) was calculated as: portal pressure/(portal vein blood flow/liver weight). Blood pressures and flows were registered on a multichannel computer-based recorder (PowerLab; ADInstruments, Colorado Springs, Colorado, USA). The external zero reference point was placed at the midportion of the animal. Hemodynamic data were collected after a 20-min stabilization period.

Immediately after the *in vivo* hemodynamic study, liver vascular responses were assessed in the isolated, *in situ* liver perfusion system, as described^42^. Livers were perfused at a constant portal flow of 35 mL/min and after 20 minutes of stabilization, liver endothelial function was evaluated analyzing endothelium-dependent vasorelaxation to incremental doses of acetylcholine (Ach; 10^−7^ to 10^−5^ M) after pre-constriction with methoxamine (10^−4^ M).

Aliquots of the perfusate were sampled for the measurement of transaminases and lactate dehydrogenase (LDH) using standard methods at the Hospital Clinic of Barcelona’s CORE laboratory. At the end of the study, liver samples from lobules that suffered WIR were stored for molecular analysis as described below.

### Liver cells isolation and treatments

Hepatocytes, Kupffer cells and liver sinusoidal endothelial cells (LSEC) were isolated from control rat livers by collagenase digestion followed by percoll gradient^43^.

Hepatocytes and Kupffer cells were treated with simvastatin (5 μM) or its vehicle for 24 h. LSEC were treated with simvastatin (1 μM) or its vehicle 1 h before undergoing 1 h anoxia using a BD Gaspak^TM^ System followed by 24 h reoxygenation.

### Von Willebrand Factor and P-Selectin immunohistochemistry

Liver samples were fixed in 10% formalin, embedded in paraffin and sectioned. After antigen retrieval procedure and endogenous peroxidase activity inhibition, sections were incubated with anti-von Willebrand Factor (1:400; Dako, Glostrup, Denmark) or anti-P-selectin (1:400; Biovision, Milpitas, CA) 1 h at room temperature. HRP-Rabbit/Mouse (Dako) secondary antibody was added. Colour development was induced by incubation with a DAB kit (Dako) and the sections were counterstained with hematoxylin. Sections were dehydrated and mounted. The specific staining was visualized and images were acquired using a microscope equipped with a digital camera and the assistance of Axiovision software. vWF relative volume was determined by point-counting morphometry on immunoperoxidase-stained sections, using a point grid to obtain the number of intercepts over vWF positive cells over the tissue. Six fields were counted in each liver. All measurements were performed by two independent blinded observers. The relative volume was calculated by dividing the number of points positive in sinusoidal areas by the total number of points over liver tissue.

### Western Blotting

Liver samples were processed and western blot performed as described^42^. Used primary antibodies: KLF2 (Santa Cruz Biotech, Santa Cruz, CA), phosphorylated eNOS at Ser1177 (Cell Signaling, Danvers, MA), and total eNOS (BD Transduction Laboratories, Lexington, KY), all 1:1000. Blots were revealed by chemiluminescence and protein expression was determined by densitometric analysis using the Science Lab 2001, Image Gauge (Fuji Photo Film, Düsseldorf, Germany). Blots were also assayed for β-actin (Sigma-Aldrich) content as standardization of sample loading.

### Superoxide and nitric oxide bioavailability

*In situ* O_2_^−^ levels were assessed in LSEC and liver tissue with the oxidative fluorescent dye dihydroethidium (DHE 10 μM; Molecular Probes Inc., Eugene, OR) as described^44^,^33^. Fluorescence images were obtained with a fluorescence microscope (Olympus BX51, Tokyo, Japan), and quantitative analysis of at least eight images per condition was performed with Image J 1.44 m software (National Institutes of Health, Bethesda, MD).

Levels of cGMP, a marker of NO bioavailability, were analyzed in liver homogenates using an enzyme immunoassay (Cayman Chemical Co., Ann Arbor, MI) as previously described^4^.

### Nitrotyrosine fluorohistochemistry

Quantitative tyrosine nitration detection was performed as previously described^45^,^22^. Briefly, slides were deparaffinized, hydrated, incubated with aqueous sodium dithionite solution (10 mM) for 10 min, washed with distilled water and then incubated overnight at 4 °C with an equimolar solution of AlCl_3_ and salicylaldehyde (200 mM). Afterwards, the aqueous solution was removed and sections were mounted in Fluoromount G medium (Southern Biotech, Birmingham, AL). Negative and positive internal controls were included. Fluorescence images were obtained with a fluorescence microscope and quantitative analysis of at least six images per sample was performed with Image J 1.44 m software.

### Neutrophil infiltration

For the evaluation of neutrophil infiltration, paraffin embedded slides were stained for specific esterase activity using a commercial available kit (Naphthol AS-D Chloroacetate Kit, Sigma-Aldrich) following manufacturer’s instructions. Six images per preparation were obtained and positive cells per field were counted.

### VCAM-1 and CD68 immunofluorochemistry

Liver cryosections of 6 μm thickness were fixed with acetone at −20 °C for 10 min, incubated with anti-VCAM-1 (1:150; BD Biosciences) 2 h at room temperature and incubated with secondary antibody Alexa Fluor 555 (1:300; Life Technologies, Alcobendas, Madrid, Spain) and 4′,6-diamino-2-fenilindol (1;3000; DAPI, Sigma-Aldrich) and mounted in Fluoromount G medium.

For CD68 detection paraffin embedded samples were used. After antigen retrieval procedure, sections were incubated with anti-CD68 (1:100; BioRad, El Prat de Llobregat, Barcelona, Spain) 1 h at room temperature and incubated with secondary antibody and mounted as described before.

Ten images per sample were obtained with a fluorescence microscope and percentage of positive area (VCAM-1) or positive cells per field (CD68) were quantified.

### Cell death

Terminal deoxynucleotidyl transferase dUTP nick end labelling (TUNEL) was performed in deparaffined liver sections using an *In Situ* Cell Death Detection Kit (Roche Diagnostics, Sant Cugat del Valles, Barcelona, Spain) according to the manufacturer’s instructions.

### Statistical Analysis

Statistical analyses were performed with the IBM SPSS Statistics 19 for Windows statistical package. All results are expressed as mean ± standard error of the mean. Comparisons between groups were performed with analysis of variance followed by LSD post-hoc test, or with Student’s *t* test when adequate. Differences were considered significant at p < 0.05.

## Additional Information

**How to cite this article**: Hide, D. *et al.* Effects of warm ischemia and reperfusion on the liver microcirculatory phenotype of rats: underlying mechanisms and pharmacological therapy. *Sci. Rep.* **6**, 22107; doi: 10.1038/srep22107 (2016).

## Supplementary Material

Supplementary Information
