# Supplementary material for: Effects of warm ischemia and reperfusion on the liver microcirculatory phenotype of rats: underlying mechanisms and pharmacological therapy
Source: Sci Rep. 2016 Feb 24;6:22107. doi: 10.1038/srep22107 (PMC4764954; doi:10.1038/srep22107)
Supplement: Supplementary Information [file srep22107-s1.pdf]

Effects of warm ischemia and reperfusion on the liver microcirculatory phenotype of rats: underlying mechanisms and pharmacological therapy

Diana Hide[1], Martí Ortega-Ribera[1], Juan-Carlos Garcia-Pagan[1], Carmen Peralta[2], Jaime Bosch[1], Jordi Gracia-Sancho[1]

1- Barcelona Hepatic Hemodynamic Lab. IDIBAPS Biomedical Research Institute – Hospital Clinic de Barcelona – CIBEREHD. Barcelona, Spain.

2- IDIBAPS & CIBEREHD.

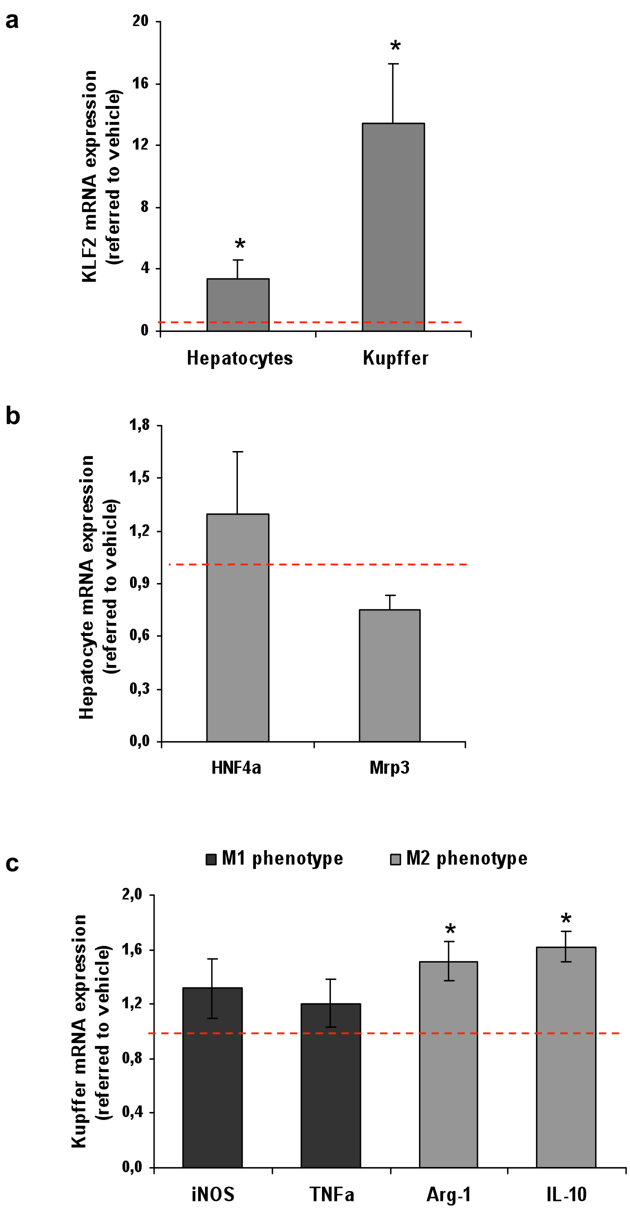

Supplementary Figure S1. Simvastatin up-regulates KLF2 expression and improves the phenotype of hepatic cells *in vitro*. (a) KLF2 mRNA expression in primary rat hepatocytes and Kupffer cells after 24h of simvastatin treatment. **(b)** Expression of hepatocyte phenotype markers in cells described in a. **(c)** Expression of Kupffer cells phenotype markers in cells described in a. Values normalized to its vehicle group (red dotted line). (n=6 per group, *p<0.05 vs. vehicle).
